# The histone lysine methyltransferase SETD8 regulates angiogenesis through HES-1 in human umbilical vein endothelial cells

**DOI:** 10.1038/s41598-020-69103-x

**Published:** 2020-07-21

**Authors:** Dong Kyu Choi, Young Kyu Kim, Sang Wook Park, Heejin Lee, Seul Lee, Sang A. Kim, Soo Jin Kim, Junyeop Lee, Wanil Kim, Sang-Hyun Min, Ji Hoon Yu

**Affiliations:** 1 New Drug Development Center, DGMIF, 80 Chumbok-ro, Dong-gu, Daegu, Republic of Korea; 2 School of Life Sciences and Biotechnology, BK21 Plus KNU Creative BioResearch Group, Kyungpook National University, Daegu, Republic of Korea; 3 Yeungnam University College of Medicine, Nam-Gu, Daegu, Republic of Korea; 4 Department of Ophthalmology, Asan Medical Center, College of Medicine, University of Ulsan, Seoul, Republic of Korea; 5 Department of Cosmetic Science and Technology, Daegu Haany University, Gyoengsan-si, Gyeongsangbuk-Do Republic of Korea

**Keywords:** Tumour angiogenesis, Mechanism of action, Epigenetics

## Abstract

Histone modifications, including histone lysine methylation, regulate gene expression in the vasculature, and targeting tumor blood vessels through histone modification decreases tumor growth. SETD8, a methyltransferase that catalyzes the mono-methylation of histone H4 lysine 20 is known to promote tumorigenesis in various cancers and its high levels of expression are related to poor prognosis. However, the detailed mechanisms by which SETD8 stimulates tumor progression and angiogenesis are still not well understood. Recent studies have demonstrated that, in vitro, BVT-948 efficiently and selectively suppresses SETD8 activity and histone methylation levels. In this study, we showed that BVT-948-mediated SETD8 inhibition in HUVECs results in an inhibition of angiogenesis. Inhibition of SETD8 not only inhibited angiogenesis but also disrupted actin stress fiber formation and induced cell cycle arrest at S phase. These effects were accompanied by increased HES-1 expression levels, decreased osteopontin levels, and a decreased differentiation of human induced pluripotent stem cells into endothelial cells. Interestingly, BVT-948 treatment reduced pathological angiogenesis in mouse OIR model. These data illustrate the mechanisms by which SETD8 regulates angiogenesis and may enable the use of a SETD8 inhibitor to treat various pathological conditions that are known to be associated with excessive angiogenesis, including and tumor growth.

## Introduction

Angiogenesis, the formation of new blood vessels, plays an essential role in not only physiological and developmental processes but also in the progression of tumor growth and other pathological conditions^1^. In tumors, endothelial cells undergo dynamic remodeling in order to deliver oxygen and nutrients to support growth. Thus, targeting angiogenesis has been proposed as a strategy to decrease tumor growth by blocking the supply of nutrients.

Although several treatments, including recombinant humanized antibodies targeting VEGF-A, have been clinically approved as anti-angiogenesis therapies and have been used in the treatment of several cancers including breast, colorectal, and lung cancers, most patients eventually experience disease relapse and there is a debate as to whether or not anti-angiogenic therapies may increase invasiveness and metastasis of tumors^2^. Therefore, new anti-angiogenesis targets are needed to overcome these problems.

Epigenetic modifications of histone are essential for physiological development and for tissue-specific gene expression. In addition, histone modifications are not only known as a marker of certain cancer but also regulate the expression of a variety of genes in the vasculature^3^. Several studies have shown that targeting the epigenetic machinery of a tumor’s blood vessels decreases tumor growth^4^. Among these components, histone methyltransferases/histone demethylases (HMTs/HDMTs) have been shown to regulate tumor angiogenesis^5^. Aberrations in their expression in pathological angiogenesis have highlighted them as potential targets for the development of therapeutics.

The methylation of lysine residues on histone or non-histone proteins by protein lysine methyltransferases (PKMTs) has a number of different biological outcomes^4,6^. Among the more than 50 PKMTs encoded by the human genome, SETD8/KMT5A is the PKMT known for the mono-methylation of histone H4 lysine 20 (H4K20me1)^7^. Through H4K20me1, SETD8 is known to participate in cell cycle progress and its suppression leads to cell cycle defects^8^.

SETD8 can also methylate non-histone targets such as proliferating cell nuclear antigen (PCNA)^9^, p53, and the p53-stabilizing factor Numb. Methylation of p53 at lysine 382 leads to decreased apoptosis either through decreased transcriptional activation by antagonizing p53 acetylation, or by promoting p53 ubiquitination for degradation^10^. The mono-methylation of PCNA at lysine 248 by SETD8 promotes tumorigenesis^11^, indicating a role for SETD8 in tumorigenesis. SETD8 also promotes the epithelial to mesenchymal transition (EMT) in cancer through its interaction with TWIST, a master regulator of the EMT^12^ thereby regulating metastasis and invasion. These reports therefore also support a role for SETD8 in tumor migration and invasion. However, the role of SETD8 in angiogenesis has not been studied in detail.

Nevertheless, there are reports that reduced *SETD8* expression levels caused by the polymorphism rs16917496 T>C are associated with a decreased susceptibility to different types of cancer, including breast and ovarian cancer, small cell lung carcinoma (SCLC), hepatocellular carcinoma (HCC), non-small cell lung carcinoma NSCLC, and childhood acute lymphoblastic leukemia (ALL)^11^. For that reason, controversial role of SETD8 need to be further investigated.

In this study, we hypothesized that SETD8 may play a critical role in pathological angiogenesis. We investigated the role of SETD8 in human umbilical vein endothelial cells (HUVECs) using the SETD8 specific inhibitor BVT-948^13^. We found that inhibition of SETD8 strongly inhibits angiogenesis and this effect was mediated by multiple mechanisms. Our findings suggest the possibility of SETD8 as a promising target for inhibiting pathological angiogenesis.

## Results

### Treatment with BVT-948 inhibits angiogenesis

Angiogenesis, the formation of new blood vessels, plays an important role in many pathological conditions including tumor growth and diabetic retinopathy. Vessels migrate and generate a new vascular network that is capable of supplying oxygen and nutrients. In endothelial cells, it has been reported that diverse histone methyltransferases (HMTs) promote proliferation, invasion, and sprouting during angiogenesis. Furthermore, HMTs have been implicated in tumor angiogenesis and their expression is related with a poor clinical diagnosis^14^.

**Figure 1:**
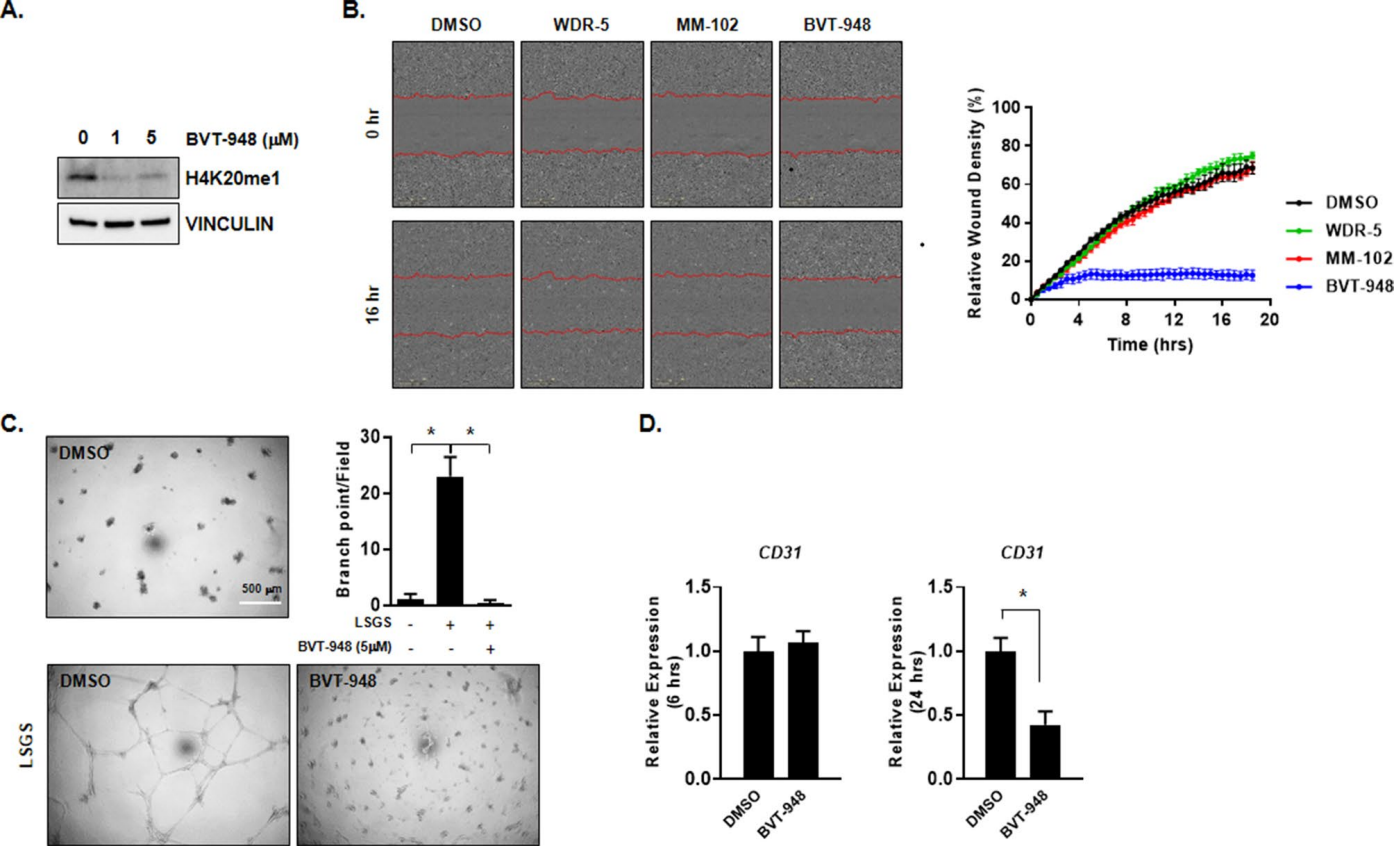
SETD8 inhibition by BVT-948 decreases endothelial cell migration and tube formation in HUVECs. (**A**) Representative western blot images showing the expression of H4K20me1 after BVT-948 (1 and 5 μM) treatment for 24 h. (**B**) Left: Phase contrast images showing a HUVEC wound healing assay after treatment with 5 μM WDR-5, 5 μM MM-102, or 5 μM BVT-948 for 16 h. DMSO was used as the control. Right: Relative wound density (%) was measured using Incucyte ZOOM software in real time manner. (**C**) Representative phase contrast images showing tube formation by HUVECs after treatment with DMSO or BVT-948 (5 μM) for 12 h. The number of branch points in a field was defined as the point of contact of three or more endothelial tubes. *p < 0.01 versus control. (**D**) HUVECs were incubated with BVT-948 (5 μM) for 6 h or 24 h and the relative expression level of *CD31* was assessed. The expression of *CD31* was normalized to that of *GAPDH*. Data are presented as the mean ± S.D. of three independent experiments. *p < 0.01 versus DMSO control.

To determine the effect of SETD8 suppression on angiogenesis, we utilized BVT-948 to inhibit the activity of SETD8. In HUVEC, 1 μM and 5 μM BVT-948 treatment reduced mono-methylation of histone 4 lysine 20 which is mediated by SETD8 (Fig. 1A), whereas BVT-948 treatment failed to reduce methylation of H3K27 (Supplementary Fig. 2). To evaluate the effect of BVT-948 on endothelial migration, scratched HUVECs were incubated with 5 μM BVT-948 and found that BVT-948 treatment dramatically inhibited HUVEC migration (Fig. 1B). Because mixed lineage leukemia (*MLL*) family proteins also possess the set catalytic domain in their carboxyl terminus and are known to play a role in angiogenesis^14^, we also used the MLL1 specific inhibitors MM-102 and WDR-5 to rule out the possibility that the BVT-948 mediated inhibition of HUVEC migration was a result of MLL inhibition. Although, we found that BVT-948 also inhibits the activity of MLL family members (MLL1, 2, 3, and 4) (unpublished data), both 5 μM MM-102 and 5 μM WDR-5 failed to inhibit HUVEC migration (Fig. 1B). These results indicate that the BVT-948 mediated inhibition of angiogenesis mainly arises as a result of the suppression of SETD8.

When HUVECs were grown on Matrigel, BVT-948 completely blocked endothelial tube formation and the number of branch points per field was also reduced (Fig. 1C). In addition, the mRNA expression levels of the pan-endothelial cell marker, *CD31*, also known as platelet endothelial cell adhesion molecule (*PECAM-1*) were reduced 24 h after BVT-948 treatment (58% ± 0.1%, compared with the DMSO control) (Fig. 1D). These results indicate that BVT-948-mediated SETD8 suppression inhibits angiogenesis in HUVECs.

### Treatment of BVT-948 disrupts actin dynamics

**Figure 2:**
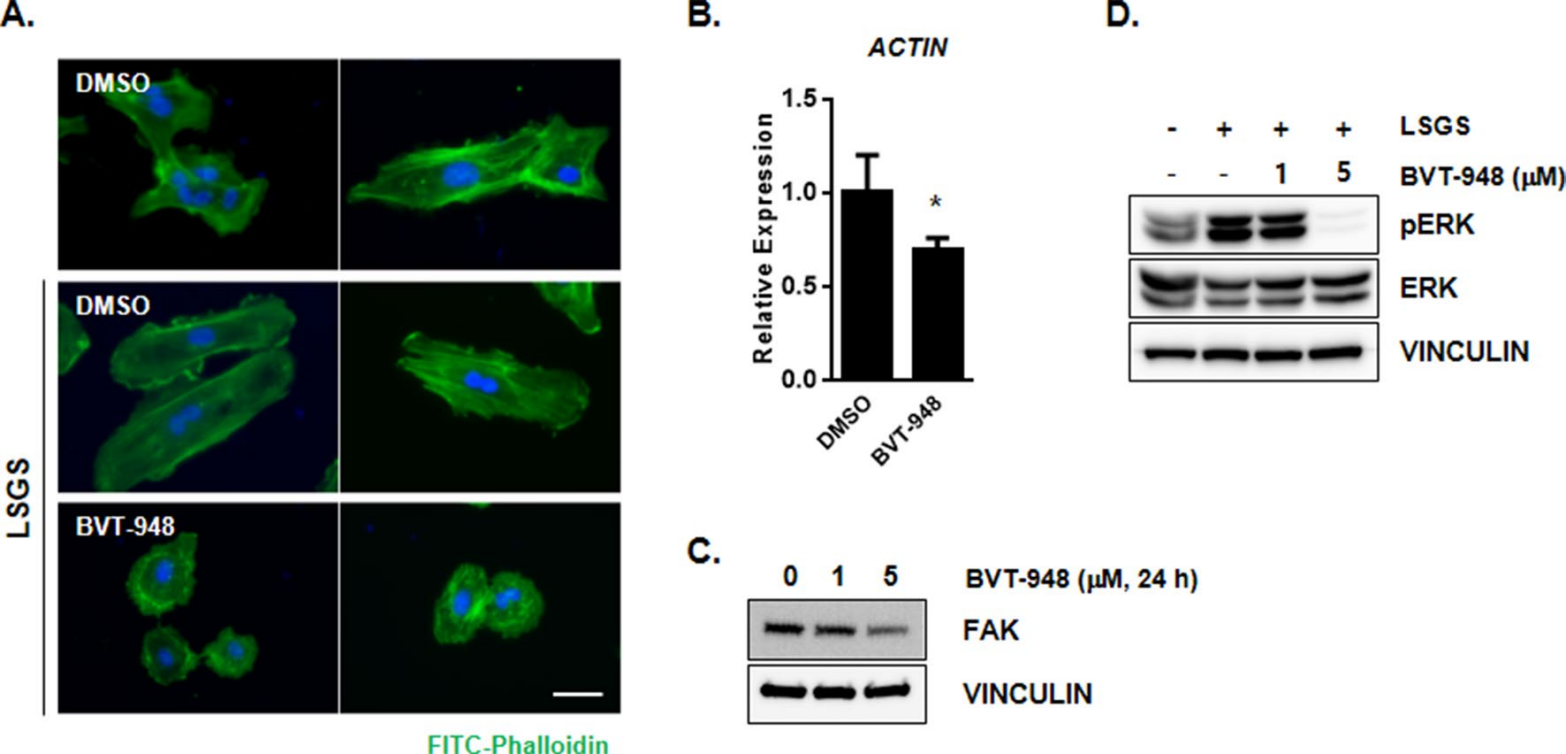
BVT-948 treatment disrupts actin stress fiber in HUVECs. (**A**) Immunofluorescence image showing actin stress fiber. HUVECs were treated with BVT-948 (5 μM) in the presence of LSGS supplement for 24 h and stained with FITC-conjugated phalloidin. Scale bars = 50 μm (**B**) Relative expression of Actin in HUVECs after 5 μM BVT-948 treatment for 24 h. (**C**) Immunoblotting showing the expression of focal adhesion kinase (FAK) in HUVECs treated with various concentration of BVT-948 for 24 h. (**D**) Immunoblots showing the levels of phospho-ERK, ERK, and vinculin in HUVECs treated with BVT-948 for 24 h. The membrane was probed with anti-phospho ERK antibody. Then the membrane was reprobed with anti-ERK antibody after stripping.

It is known that the assembly of actin polymers is an absolute requirement for cell migration^15,16^. Gerhardt et al. have reported that the migrating endothelial tip cell during retinal angiogenesis is positive for F-actin expression^17^. To compare migrating endothelial cell phenotype using polymerized F-actin, we stained HUVECs with FITC-conjugated phalloidin. Following treatment with the DMSO control, HUVECs contained a well-organized F-actin leading edge and exhibited lamellipodia. However, HUVECs treated with BVT-948 showed a round phenotype and a disrupted F-actin organization. Interestingly, there were no significant change in the roundness of the nuclei suggesting that BVT-948 has no toxicity in cultured endothelial cells (Fig. 2A). BVT-948 treatment also significantly decreased actin mRNA levels, consistent with the proposed role for SETD8 in controlling actin dynamics (Fig. 2B).

Consistent with the loss of actin stress-fibers, SETD8 inhibition also resulted in decreased expression levels of focal adhesion kinase (*FAK*) (Fig. 2C). FAK not only provides an adhesion link between actin and the extracellular matrix in the cell membrane but also transmit various cellular signals through Src-dependent phosphorylation and is therefore very important in regulating cell migration. *FAK*-deficient fibroblasts have been shown to have a disrupted F-actin assembly phenotype^18,19^. Here, we found that FAK-mediated phosphorylation of ERK was reduced after BVT-948 treatment (Fig. 2D), although the phosphorylation of AKT was not altered (unpublished data). These results show the involvement of FAK signaling and actin dynamics in the BVT-948 mediated inhibition of angiogenesis.

### Treatment of BVT-948 moderately reduces cell proliferation

**Figure 3:**
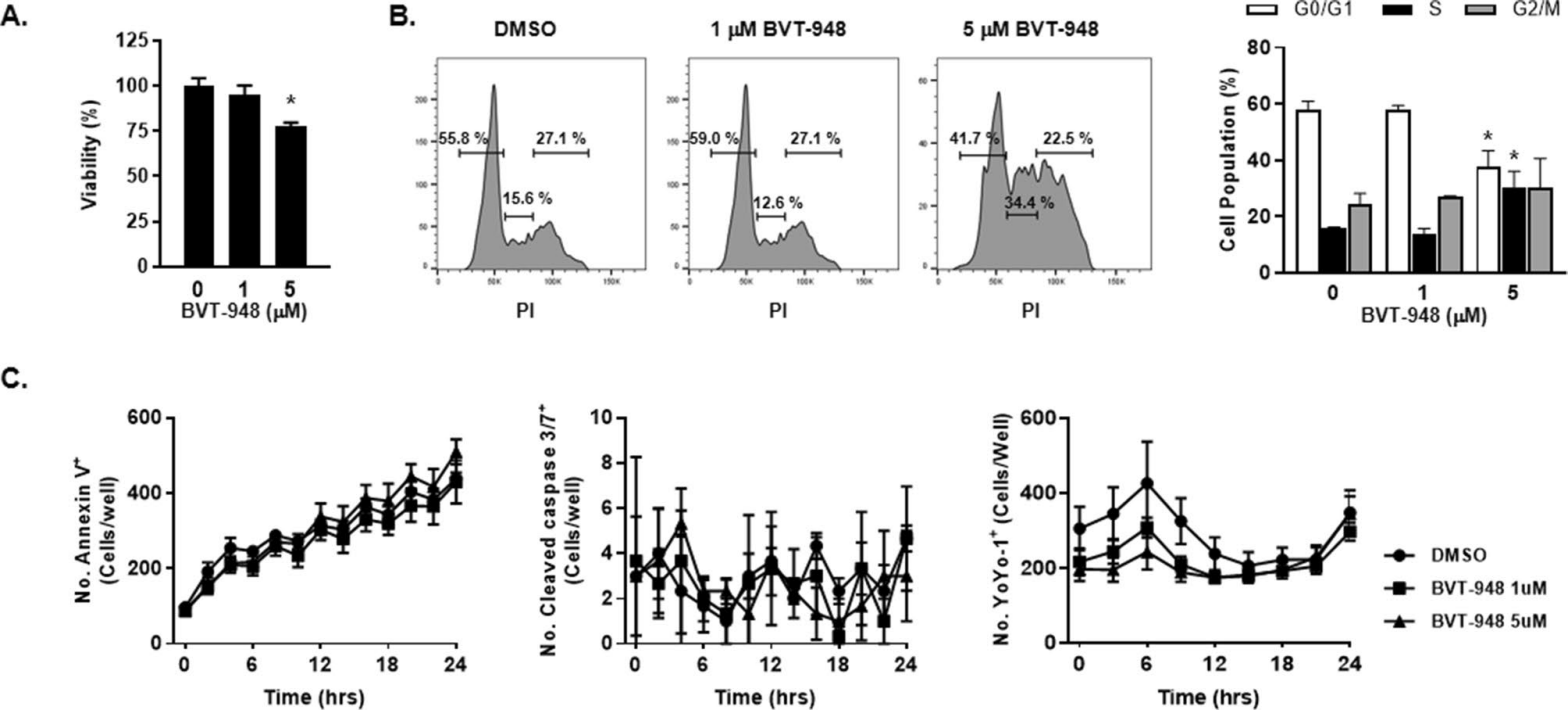
BVT-948 treatment decreases the proliferation of HUVECs. (**A**) Cells were treated with 1 or 5 μM BVT-948 for 24 h and cells viability was measured using CCK-8. *p < 0.01 versus control. (**B**) Cells were treated with 1or 5 μM BVT-948 for 24 h and cell cycle was analyzed after PI staining. *p < 0.01 versus control. (**C**) Various cell cytotoxicity monitoring dyes were used to assess cytotoxicity in BVT-948 (1 and 5 μM) treated HUVECs and number of positive cells were counted using the Incucyte ZOOM system after 24 h of treatment. Left: Number of annexin V^+^ cells. Middle: Number of cleaved caspase 3/7^+^ cells. Right: Number of YoYo-1^+^ cells. Data are presented as the mean ± S.D of three independent experiments.

To assess whether the decreased angiogenesis was a result of altered cell viability, we measured cell proliferation, cell cycle, and cytotoxicity. Accordingly, the cell viability assay using CCK-8 revealed that, compared to the control, 5 μM BVT-948 treatment of HUVECs for 24 h decreased cell viability by 23%, whereas 1 μM BVT-948 treatment did not (Fig. 3A). In addition, 5 μM BVT-948 treatment caused cell cycle arrest at S phase. After 24 h of treatment, 5 μM BVT-948 induced cell cycle arrest at S Phase (15.6% in DMSO control vs. 34.4% in the BVT-948 treated cells), whereas the population of G0/G1 was moderately reduced (Fig. 3B). In addition, we analyzed DNA replication of HUVECs treated with BVT-948 using EdU incorporation assay. And found that 5 μM BVT-948 treatment resulted in an almost complete absence of EdU incorporation, showing lacking of DNA synthesis in these cells (Supplementary Fig. 3).

However, when we applied the cell impermeable nucleic acid dye, YoYo-1, we failed to detect any difference in signals. Similarly, the number of cleavage caspase 3/7+ or Annexin V+ cells were not significantly changed by BVT-948 treatment (Fig. 3C). These data indicate that BVT-948 causes a moderate reduction in cell proliferation and cell cycle arrest in an important mechanism. Interestingly, decreased phosphorylation of ERK also decreases HUVEC proliferation. Therefore, this decreased proliferation may partially contribute to the BVT-948 mediated suppression of angiogenesis.

### Treatment of BVT-948 increases expression of HES1

**Figure 4:**
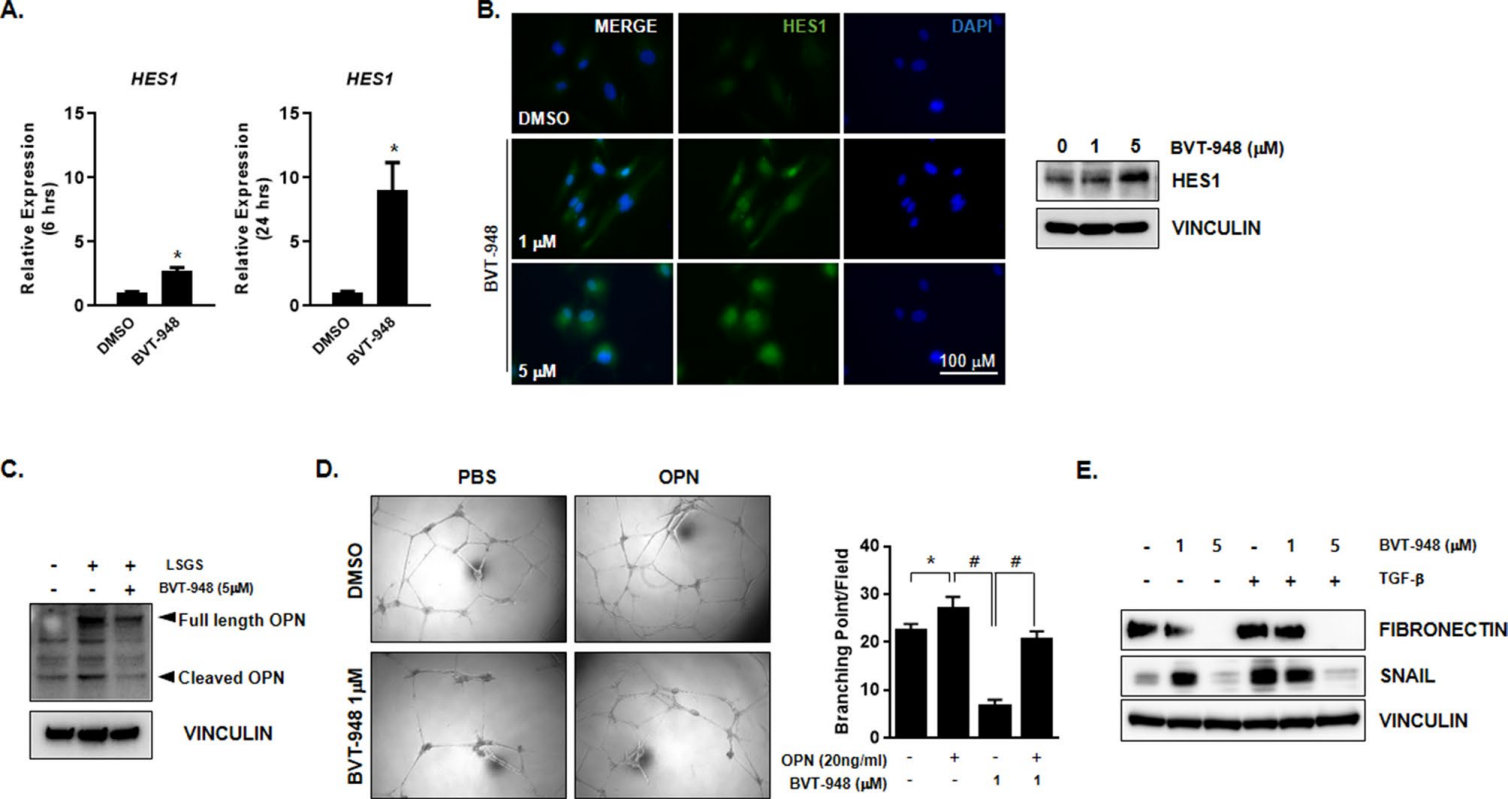
BVT-948 induced inhibition of angiogenesis is mediated through decreased osteopontin expression. (**A**) The relative expression of *HES-1* in HUVECs treated with 5 μM BVT-948 was measured by real-time quantitative PCR. *p < 0.01 versus DMSO control. (**B**) Left: immunofluorescence images showing the expression of HES-1 in HUVECs. Right: Representative western blot images showing the expression of HES-1 HUVECs treated with various concentrations of BVT-948. (**C**) Western blot showing the expression levels of osteopontin in HUVECs treated or not with 5 μM BVT-948 in the presence or absence of LSGS supplement. (**D**) Phase contrast images showing HUVEC tube formation. HUVECs were treated with 1 μM BVT-948 treated in the presence or absence of osteopontin (20 ng/mL) and the number of branch points in a given field was counted. Data are presented as the mean ± S.D of three independent experiments. *p < 0.05; #p < 0.05 versus control. (**E**) Western blot image showing the expression levels of FN1 and SNAIL in HUVECs treated or not with 5 μM BVT-948. TGF-β or PBS was treated after 30 min of BVT-948 treatment.

To evaluate the signaling pathways important for the BVT-948 mediated inhibition of angiogenesis, we analyzed the expression of angiogenesis related genes and found that BVT-948 treatment for either 6 h or 24 h increased the mRNA expression of hairy/enhancer of split homologue-1 (*HES-1*) in HUVECs 2.7 fold and 9.02 fold, respectively, relative to DMSO treatment (Fig. 4A). Recently, Xing-Xing et al. have reported that HES-1, which is downstream of NOTCH1, suppresses VEGF-induced angiogenesis by down-regulating osteopontin (OPN). In that study, they showed that decreased HES-1 in endothelial cells within atherosclerosis plaque region resulted in an increase in OPN levels followed by increased angiogenesis.

Immunocytochemistry and western blotting also showed that HES-1 expression was increased after 24 h of BVT-948 treatment (Fig. 4B). Our results are in agreement with previous reports showing that suppression of SETD8 during erythroid maturation increases *HES-1* expression^20^. Nevertheless, the expression of OPN in HUVECs was decreased following BVT-948 treatment (Fig. 4C). OPN, also known as secreted phosphoprotein1, is an acidic glycoprotein and a member of the small integrin-binding N-linked glycoprotein family. OPN plays many roles in the pathogenesis of many diseases. Previous reports have also shown that OPN stimulates angiogenesis via the avβ3/PI3-K/AKT/eNOS/NO signaling pathway^21^. Here, the tube formation inhibited by BVT-948 treatment was rescued by treatment of HUVECs with 20 ng/mL recombinant human OPN (Fig. 4D). These results strongly indicate that the decreased OPN levels caused by BVT-948 treatment are very important in inhibiting angiogenesis. Next, we wanted to further understand the mechanism by which SETD8 regulated angiogenesis, Because, during tumor angiogenesis, several angiogenic stimuli induce the expression of epithelial- mesenchymal transition (EMT) related genes including *SNAIL* and *FIBRONECTIN*, we hypothesized that SETD8 may regulates the expression of EMT related genes. Therefore, the expression of these genes contributes to the more highly motile endothelial cell phenotype facilitating the invasion and metastasis of tumor cells^22^. Interestingly, it is also known that SETD8 promotes the EMT in cancer through its interaction with TWIST^12^. Because the release of latent TGF-βfrom degraded ECM transduces the EMT signaling cascade, we treated HUVECs with or without BVT-948 in combination with TGF-β and measured the expression of EMT-related proteins to evaluate the role of SETD8 during EMT.

In our study, BVT-948 decreased the expression levels of FIBRONECTIN and SNAIL after 24 h of treatment (Fig. 4E). TGF-β stimulates SNAIL expression through RTKs^23^, and SNAIL coordinates the histone modification of EMT-related genes by binding to the promoter region of these genes. Thus, SNAIL activates the EMT during development, fibrosis, and cancer^24^. In addition, the expression of FIBRONECTIN, a component of the mesenchymal compartment, is also associated with the invasive and metastatic phenotype of various tumors^25^. The up-regulation of these proteins is tightly related with the expression of *N-cadherin*, FAK-mediated cell migration, and invasion in MCF-7 cells. These results therefore suggest a role for SETD8 during the EMT process.

### Treatment with BVT-948 inhibits the angiogenesis during development and pathological conditions

**Figure 5:**
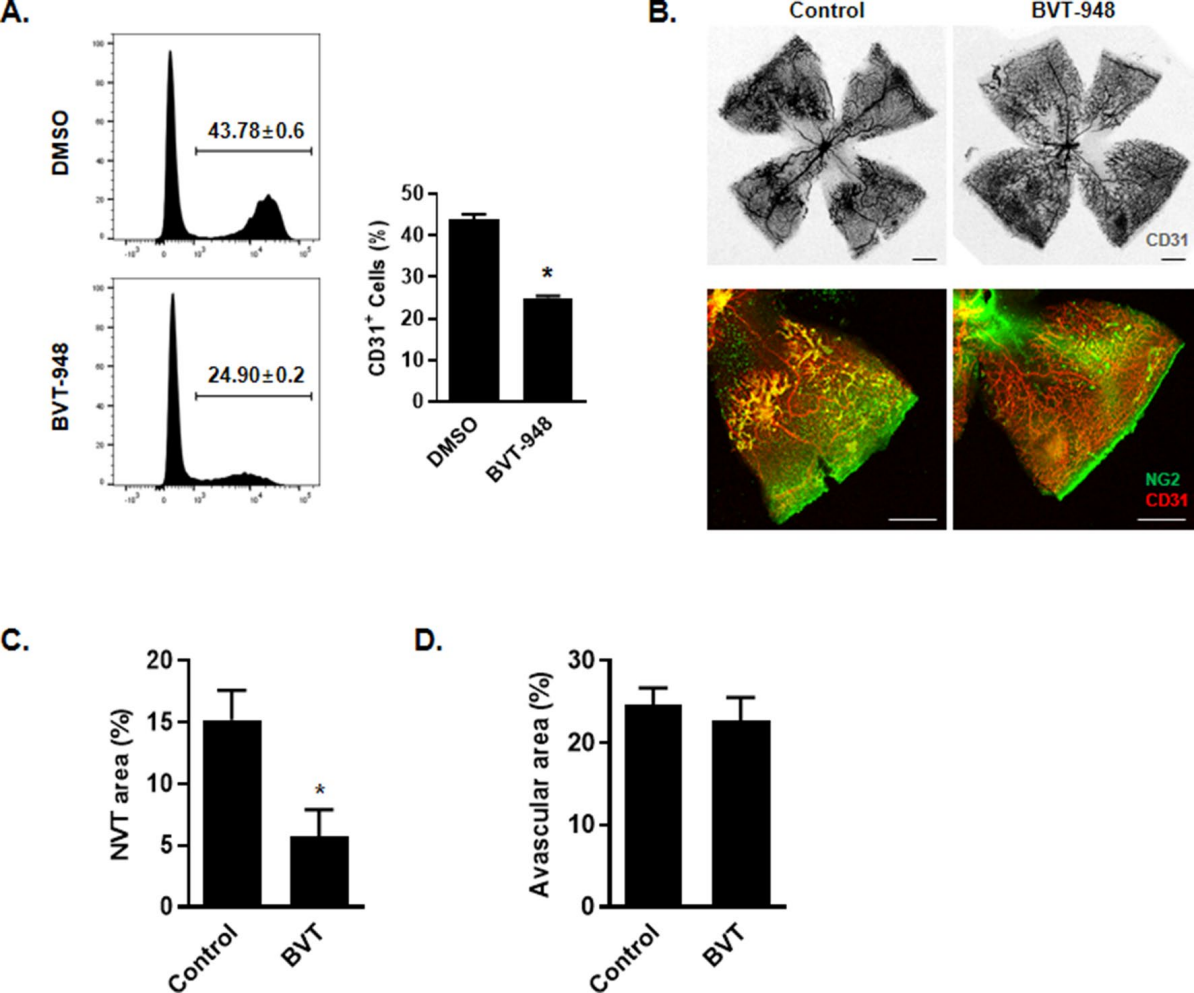
BVT-948 suppresses developmental and pathological angiogenesis. (**A**) Representative FACS analysis of the number of CD31+ endothelial cells in differentiated human iPSCs. Human iPSCs were differentiated into endothelial cells for 3 days in the presence of either DMSO or BVT-948 (5 μM). The percentage of the pan-endothelial cell marker CD31 expressing cells was measured by flow cytometry *p < 0.01 versus DMSO control. (**B**) Representative images showing vascular network in the retina of OIR mice. Mice were treated with BVT-948 for 5 days and retinas were immunostained with CD31 and NG2, Scale bars = 100 μm. (**C**, **D**) Comparisons of CD31+ blood vessels and NG2+ neovascular tufts (NVT) in OIR model. (Each group, n = 10) *P < 0.01 versus control.

For further determination of the role of SETD8 in angiogenic activity during development, we employed an endothelial cell differentiation model using human induced pluripotent stem cells. When basic FGF is withdrawn from the growth medium of these cells, the human iPSCs can differentiate into all germ layers, thereby providing a powerful tool for assessing developmental biology^26^. In the presence of VEGF-A, human iPSCs differentiated into endothelial cells such that 43% of the cells expressed the pan-endothelial cell marker, CD31, on their surface. However, when BVT-948 was continuously present in the medium during differentiation, the percentage of CD31 expressing cells was decreased by 20% (Fig. 5A). Furthermore, when *SETD8* RNP transfected hiPSCs were differentiated into vascular endothelial cell, the population of CD31+/CD144+ endothelial cell was moderately decreased (Supplementary Fig. 1). Taking advantage of the role of SETD8 in developmental angiogenesis model, we further evaluated the role of SETD8 inhibition in pathological condition using oxygen induced retinopathy (OIR) model. The OIR model mimics human retinopathy of premature and certain aspect of proliferative diabetic retinopathy and is a useful system for studying ophthalmic neovascular disease^27^. We intravitreally injected BVT-948 to the OIR model mice from P12 to P17 and examined their retinas on P17. Compared with control mice, BVT-948 treated mice displayed reduced NG2 positive neovascular tuft (NVT) area. Moreover, the vessels of BVT-948 treated mice were more densely and well networked in the central region of retina (Fig. 5B, C). However, avascular area was not different between those mice (Fig. 5D). These data indicated that SETD8 may participate in angiogenic development and pathological angiogenesis.

## Discussion

Angiogenesis plays a crucial role under normal physiological conditions. However, excessive angiogenesis is associated with many pathological conditions including arthritis, diabetes, psoriasis, and tumor growth. In tumors, endothelial cells are regulated by various stimuli such as VEGF, EGF, and bFGF which are either secreted from the endothelial cells themselves or from stromal cells^28^. It is also known that histone modification also regulates angiogenesis by altering gene expression patterns^3^. Thus, the epigenetic machinery, especially histone methyltransferases/histone demethylases (HMTs/HDMTs), may be a promising target to inhibit angiogenesis.

SETD8 is a histone methyltransferase for H4 lysine 20^7,29^. SETD8 is involved in many physiological process including the cell cycle, chromatin condensation^8^, apoptosis^10^, tumorigenesis^11^, and the epithelial to mesenchymal transition^12^.

Based on these previous reports, we hypothesized that SETD8 may play an important role in angiogenesis. Here, we showed that inhibition of SETD8 using the specific inhibitor BVT-948 inhibited not only HUVEC migration but also tube forming activity. In agreement with these findings, we found that BVT-948 also decreased the expression of the pan-endothelial cell marker, *CD31*.

Endothelial cell migration is characterized by increased actin cytoskeleton and actin remodeling including the formation of sheet-like membrane protrusions called lamellipodia and spike-like extensions called filopodia^30^. The constant remodeling of the actin cytoskeleton into filopodia, lamellipodia, and stress fibers is heavily involved in endothelial cell migration. Our results showed that inhibition of SETD8 disrupts not only filopodia but also lamellipodia at the leading edge. In addition, the expression levels of actin itself were decreased by SETD8 inhibition along with the expression levels FAK and the phosphorylation of levels of ERK. Our data are supported by reports showing a relationship between FAK and F-actin during junction restructuring at the blood-testis barrier (BTB)^31^.

During cell cycle progress, the expression of SETD8 is finely regulated by multiple E3 ligases and is sustained at a maximum level in the G2/M phase and at a minimum level at S phase^32^. Suppression of H4K20me1 through SETD8 disruption leads to cell cycle defects, chromatin de-condensation, and enlarged nuclei^8^. Intriguingly, our results revealed that the suppression of angiogenesis by SETD8 suppression was partially derived from decreased proliferation of HUVECs and cell cycle arrest at S phase. In our results, it do not seems that the increase in the S phase fraction after BVT-948 treatment represents an active replication of DNA in HUVECs, but it seems likely that cells were arrested during progression of S phase.

In HUVECs, activation of the PI3-kinase/Akt signaling pathway is mainly involved in cellular survival, whereas activation of the ERK signaling is mainly involved in cellular proliferation^33^. We showed that BVT-948 did not reduce PI3-kinase/Akt signaling, whereas it did reduce phosphorylation of ERK and these data were supported by the finding that BVT-948 treatment does not have an effect on cell cytotoxicity.

Recently, Xing-Xing et al. have shown that VEGF-A, a key angiogenic inducer, promotes angiogenesis by down-regulating *HES-1* followed by an up-regulation of *OSTEOPONTIN* in atherosclerotic plaques. It is also known that HES-1 acts as transcriptional mediator at the *OPN* promoter region and that OPN levels are increased in endothelial cells in atherosclerotic plaques^34^. In agreement with these reports, we showed that BVT-948 up-regulates the expression of *HES-1* and the BVT-948 mediated inhibition of angiogenesis could be prevented by OPN treatment. These finding suggest up-regulating *HES-1* via BVT-948 may be a promising way not only to prevent the formation of plaques in atherosclerosis but also to inhibit tumor angiogenesis.

By binding to integrin avβ3, OPN increases Rho GTPase activity via a downstream signaling pathway that involves RANKL or SRC, PYK2, and FAK in prostate cancer cells^35^, supporting our results that inhibition of SETD8 dramatically decreases OPN expression, FAK expression, actin remodeling, and migration in HUVECs.

We also showed that angiogenic endothelial cells express SNAIL and FIBRONECTIN, a crucial mediator of angiogenic sprouting, and the expression levels of these proteins were markedly decreased in HUVECs after BVT-948 treatment. In all tissues, key events in the epithelial to mesenchymal transition are increased cell motility and the expression of mesenchymal genes and the same situation is seen for the endothelial to mesenchymal transition (EndMT), which was first reported in developmental studies of heart formation^36^, with further studies demonstrating the role of SLUG, SNAIL, and TWIST during the EndMT^37^. *SLUG* expression is up-regulated in tumor associated endothelial cells^38^ and EndMT has been identified as one origin of cancer-associated fibroblasts^39^. In cancer cells, TWIST induces *N-cadherin* expression by recruiting SETD8 to its promoter^4^.

Wnt signaling induces the proliferation and migration of endothelial cells via both the canonical and non-canonical Wnt signaling pathways^40^ and Zhenfei et al. have also shown that the increased levels of H4K20me1 seen following Wnt stimulation are catalyzed by SETD8 and that SETD8 can participate in Wnt signaling in both mammalian cells and zebrafish^41^. In our unpublished data, BVT-948 treatment decreased the expression of β-catenin in HUVECs. Recently, Maggy et al. have reported that soluble E-Cadherin promotes angiogenesis via β-catenin dependent signaling and that β-catenin knockdown inhibits angiogenic tube formation^42^ and increases embryonic fibronectin expression^43^. For this reason, the relationship between decreased β-catenin and the SETD8 suppression-mediated inhibition of angiogenesis needs to be studied in the future.

During development, stem cells differentiate into various endothelial cell types and these phenotypes can be a useful tool for studying angiogenesis. To mimic embryonic differentiation, we used the human iPSC differentiation model and found that BVT-948 dramatically inhibits VEGF-A induced endothelial cell differentiation, indicating a role for SETD8 in developmental angiogenesis. In accordance with these results, we also found that BVT-948 mediated SETD8 inhibition presented beneficial effects in pathological condition, especially in OIR model.

Although our results show that BVT-948 mediated SETD8 suppression has anti-angiogenic actions through multiple mechanisms, further studies are required to clarify the mechanism. First, although we found that SETD8 suppression alters the expression levels of certain genes, unfortunately the mechanism by which SETD8 suppression regulates the expression of these genes remains unknown. For this reason, a detailed epigenomics study of angiogenesis related transcription factors after SETD8 suppression, which could explain these changes, needs to be conducted. Second, an implication of SETD8 on tumor progression needs to be studied. Song et al. and others works showed that reduced SETD8 expression contributes to the early onset of breast cancer^44^. In this aspect, role of SETD8 on certain type of cancer and its mechanism is needed to be investigated. Finally, the role of the SETD8 under pathological conditions also needs to be studied using genetically modified mice.

Here, we showed that SETD8 suppression inhibits angiogenesis through multiple mechanisms. These data partially explain how SETD8 regulates angiogenesis and may enable the use of a SETD8 inhibitor to treat various pathological conditions.

## Methods

### Cell culture and reagent

HUVECs were purchased from Lonza (Basel, Switzerland) and cultured on 0.1% gelatin-coated culture plates in endothelial cell growth basal medium (Lonza, Basel, Switzerland) supplemented with LSGS (Lonza, Basel, Switzerland) and were maintained at 37 °C in a humidified 5% CO_2_ atmosphere. Cells below passage seven were used for all experiments and the medium was changed daily. BVT-948 was purchased from TOCRIS (Bristol, UK).

### Scratched wound assays

HUVEC migration was measured using Incucyte ZOOM. Briefly, HUVECs were grown to confluency in a 96 well image Lock plate (Sartorius, GE) that had been coated with 0.1% gelatin. After 24 h, the confluent monolayers were “scratched” with a wound making kit to create a cell-free wound area and the monolayers were cultured in the presence of DMSO or BVT-948 at the indicated concentration. Phase contrast images were taken every 30 min and the relative wound density was calculated using the Incucyte ZOOM software.

### Tube forming assays

Ninety-six well tissue culture plates were coated with growth factor reduced Matrigel (Corning, NY, USA) and the Matrigel was allowed to solidify. After 30 min, HUVECs (1 × 10^4^) were seeded onto the Matrigel coated plate and medium containing either DMSO or BVT-948 was added. Image were taken after 12 h and the number of tube branches was counted.

### Cell cycle analysis

HUVECs were treated with various concentration of BVT-948 for 24hrs and washed twice with PBS. Cells were fixed using 70% cold EtOH for 1 hr and washed twice with cold PBS. After incubation with 200 μg/ml RNase A for 1 h at 37 ℃, cells were stained with 2.5 μg/ml propidium iodide for 15 min at room temperature in the dark. The samples were immediately analyzed flow cytometry and cell cycle phase distribution was determined using FlowJo software Version 10 (FlowJo, OR, USA).

### FACS analyses

Cells were harvested using 0.25% trypsin–EDTA (Invitrogen, CA, USA) and then resuspended in PBS/2% FBS at a density of 1 × 10^6^ cells per 100 μL. The cells were incubated for 30 min with PE conjugated anti CD31 antibody (BioLegend, CA, USA) or Alexa fluor 647 conjugated anti CD144 antibody (BioLegend, CA, USA) and dead cells were excluded by staining with 7-aminoactinomycin D (Invitrogen, CA, USA). Analyses were performed using a FACS Aria III (BD Bioscience, NJ, USA) and data were analyzed using FlowJo software Version 10 (FlowJo, OR, USA).

### Phalloidin staining

After compound treatment, the cultured cells were fixed with 3% paraformaldehyde (PFA) for 15 min and PBS containing 0.1% Triton X-100 was added to permeabilize the cells. The cells were washed with PBS and then blocked with 5% BSA in PBST for 1 h at room temperature. Cells were then incubated with Alexa Fluor™ 488 Phalloidin (Thermo Scientific, MA, USA) for 30 min at room temperature to visualize F-actin. Cells were then washed three times and Hoechst 33,342 was added to visualize nuclei, after which the cells were mounted in fluorescent mounting medium (DAKO). Immunofluorescent images were acquired using EVOS (Thermo Fisher Scientific, USA).

### Quantitative real-time PCR

Total RNA was extracted from cultured HUVECs using Trizol reagent (Invitrogen, CA, USA) according to the manufacturer’s instructions and a total of one microgram of total mRNA was used for cDNA synthesis using the GoScript reverse transcription system (Promega, WI, USA). Quantitative RT-PCR was performed with the indicated primers using FastStart SYBR Green Master polymerase (Roche, Basel, Switzerland) and StepOnePlus Real-Time PCR system (Applied Biosystems, CA, USA). The average threshold cycle (Ct) was determined from triplicate reactions and the levels of gene expression relative to *GAPDH* were determined.

### Western blotting

Total proteins were fractionated by SDS-PAGE and transferred onto poly-vinylidene difluoride membrane using a Trans-Blot Turbo transfer system (Bio-Rad, Hercules, CA, USA) according to the manufacturer’s protocols. After incubation with 5% nonfat milk in TBST (10 mM Tris, pH 8.0, 150 mM NaCl, 0.5% Tween 20) for 60 min, the membrane was incubated with the appropriate primary antibody at 4 °C overnight. Membranes were then washed three times with TBST for 10 min and incubated with a 1:5,000 dilution of the appropriate HRP-conjugated anti-mouse or anti-rabbit antibodies for 2 h. The blots were washed with TBST three times and immunoreactive bands developed using the clarity ECL system (Bio-Rad) and quantitated using the ImageQuant LAS4000 system (GE Healthcare). The blots were cropped from different part of the same gel otherwise indicated and full length images were available in Supplementary Figures. VINCULIN was used for loading control.

### Cell viability and caspase 3/7 activity assay

One thousand cells were seeded into each well of a 96 well plate. After 24 h, the cells were washed once in PBS and then incubated with indicated concentration of compound. Cells were incubated for 24 h with Caspase-3/7 Green apoptosis reagent (Sartorius, GE) to detect caspase cleavage and Annexin V Green reagent (Sartorius, GE) to detect early apoptosis. The Images were captured and analyzed using Incucyte ZOOM (Sartorius, GE). For the viability assay, CCK-8 was added and the optical density at 450 nm was measured. OD data were normalized with DMSO and control medium.

### hiPSC maintenance and endothelial cell differentiation

The human induced pluripotent stem cell line, hFSiPS1, was kindly provided by the National Stem Cell Bank of Korea (Korea National Institute of Health). The hFSiPS1 cells were maintained in an hESC-qualified Matrigel (Corning, NY, USA) coated dish in mTESR1 (Stem Cell Technologies, CA, USA). For endothelial differentiation, the hiPSCs were dissociated using Accutase (Stem Cell Technologies) and then seeded on Matrigel at a density of 1.5 × 10^5^ cells per well in a 6-well dish with mTESR1 containing 10 μM Y-27632 (Stem Cell Technologies). After one day, the cells were washed once with PBS, and then Stemdiff Mesoderm induction medium (Stem Cell Technologies) supplemented with 1 μM CP21R7 (Roche, Switzerland) was added. After three days, the medium was replaced by an endothelial cell induction medium consisting of StemPro-34 SFM medium (Life Technologies, USA) supplemented with 200 ng/mL VEGF-A (Peprotech, USA) and 2 μM forskolin (Sigma-Aldrich) for 2 days. After endothelial cell induction, medium was replaced with EGM-2 (Lonza) medium supplemented with 50 ng/mL VEGF165 (Peprotech) and 1 μM BMX for endothelial cell maturation.

### Animal experiments and analyses

Specific pathogen-free mice were purchased from The Jackson Laboratory (Bar Harbor, ME, USA). Animal care and experimental procedures were approved by the Animal Care Committee of Yeungnam University College of Medicine and handled according to the NIH guideline. The oxygen-induced retinopathy (OIR) mouse model was generated based on a previous report^27^. Briefly, the newborn mice at P7 and their nursing mothers were exposed to 75% oxygen in a hyperoxic chamber (O_2_ Control InVivo Cabinet, Coy Laboratory, MI, USA) for 5 days, and then were returned to room air. The anesthetized mice were injected intravitreally with 1ul of 10 mM BVT-948 or DMSO control at P12 using a micro-injector (Nanoliter 2000; World Precision Instruments, Sarasota, FL, USA) fitted with glass capillary pipettes. After 5 days, immunohistochemistry of the whole-mounted retinas was performed as previously described^45^. Briefly, the retinal cups were incubated along with hamster anti-CD31 monoclonal antibody (2H8; Millipore Corp.) and rabbit anti-NG2 polyclonal antibody (Millipore, AB5320). After several washes, the samples were incubated for 4 h at room temperature with Cy3-conjugated anti-hamster IgG antibody (Jackson ImmunoResearch Laboratories) and FITC-conjugated anti-rabbit IgG antibody (Jackson ImmunoResearch Laboratories).

### Statistical analysis

Results are presented as mean ± S.D. The significance of differences was evaluated by a one-way ANOVA, followed by a post-hoc analysis using Dunnett’s t-test, performed with the Prism software package (GraphPad Software, San Diego, CA, USA).

## Supplementary information

Supplementary file1

## Data Availability

The authors confirm that the data supporting the findings of this study are available within the article and full length images of blots in main paper are available in supplementary files.
