# Supplementary material for: The histone lysine methyltransferase SETD8 regulates angiogenesis through HES-1 in human umbilical vein endothelial cells
Source: Sci Rep. 2020 Jul 21;10:12089. doi: 10.1038/s41598-020-69103-x (PMC7374624; doi:10.1038/s41598-020-69103-x)
Supplement: Supplementary file 1 — Supplementary file1 [file 41598_2020_69103_MOESM1_ESM.docx]

**The histone lysine methyltransferase SETD8 regulates angiogenesis through HES-1 in human umbilical vein endothelial cells.**

Dong Kyu Choi^1^, Young Kyu Kim^1^, Sang Wook Park^1^, Heejin Lee^1,2^, Seul Lee^1^, Sang A. Kim^3^, Soo Jin Kim^3^, Junyeop Lee^4^, Wanil Kim^5^, *Sang-Hyun Min^1^, *Ji Hoon Yu^1^.

^1^ New Drug Development Center, DGMIF, Dong-gu, Daegu, Republic of Korea.

^2^ School of Life Sciences and Biotechnology, BK21 Plus KNU Creative BioResearch Group, Kyungpook National University, Daegu, Korea

^3^ Yeungnam University College of Medicine, Nam-Gu, Daegu, Republic of Korea

^4^ Department of Ophthalmology, Asan Medical Center, University of Ulsan, College of Medicine, Seoul, Republic of Korea

^5^ Department of Cosmetic Science and Technology, Daegu Haany University, Gyoengsan-si, Gyeongsangbuk-Do, Republic of Korea

- **Supplementary figures**
- **Supplementary material & methods**

**Supplementary figures**

**
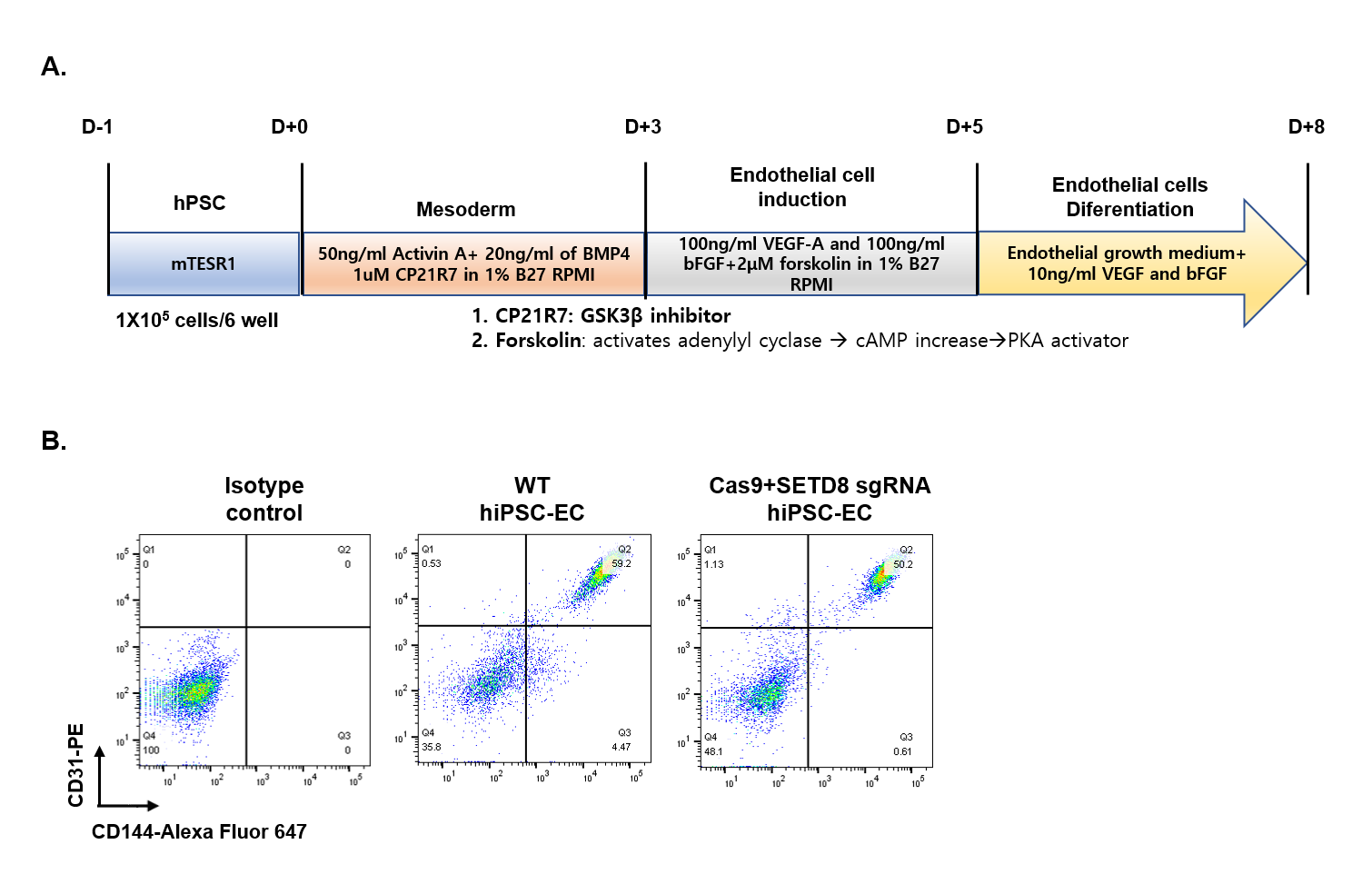
**

**Supplementary figure 1.** (A) Schematic drawing showing differentiation protocol for endothelial differentiation of hiPSc (B) Representative FACS analysis of percentage of CD31+/CD144+ endothelial cell in differentiated human iPSCs. hiPSCs were transfected with or not sgRNA targeting human *SETD8*. A method is available in supplementary material & Methods.


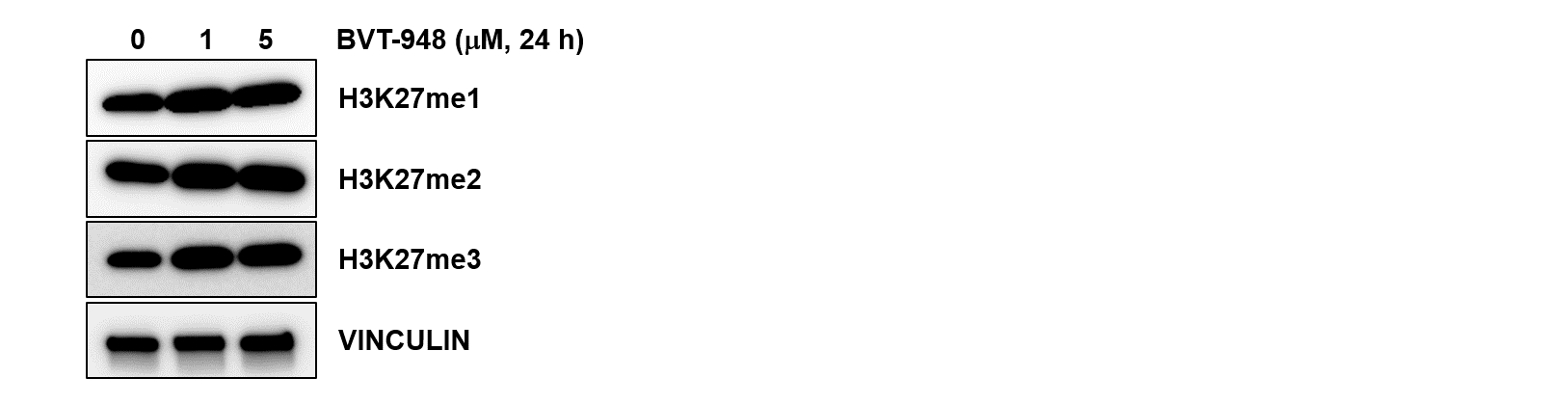


**Supplementary figure 2.** Representative western blot images showing the expression of H3K27me1, H3K27me2, and H3K27me3 after BVT-948 (1 and 5 μM) treatment for 24 h.

**
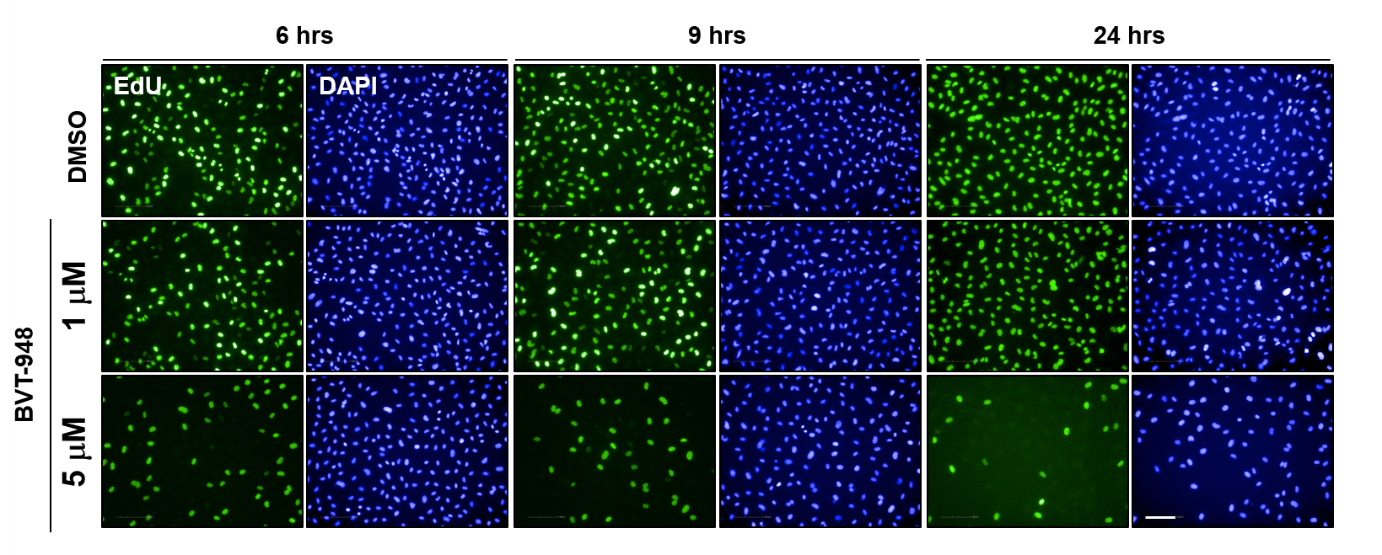
**

**Supplementary figure 3.** Immunofluorescence image showing EdU incorporation. HUVECs were treated with BVT-948 (1 or 5 μM) for 6, 9 or 24 h followed by EdU treatment for 1 h and Edu was labelled with FITC. Scale bars = 100 μm


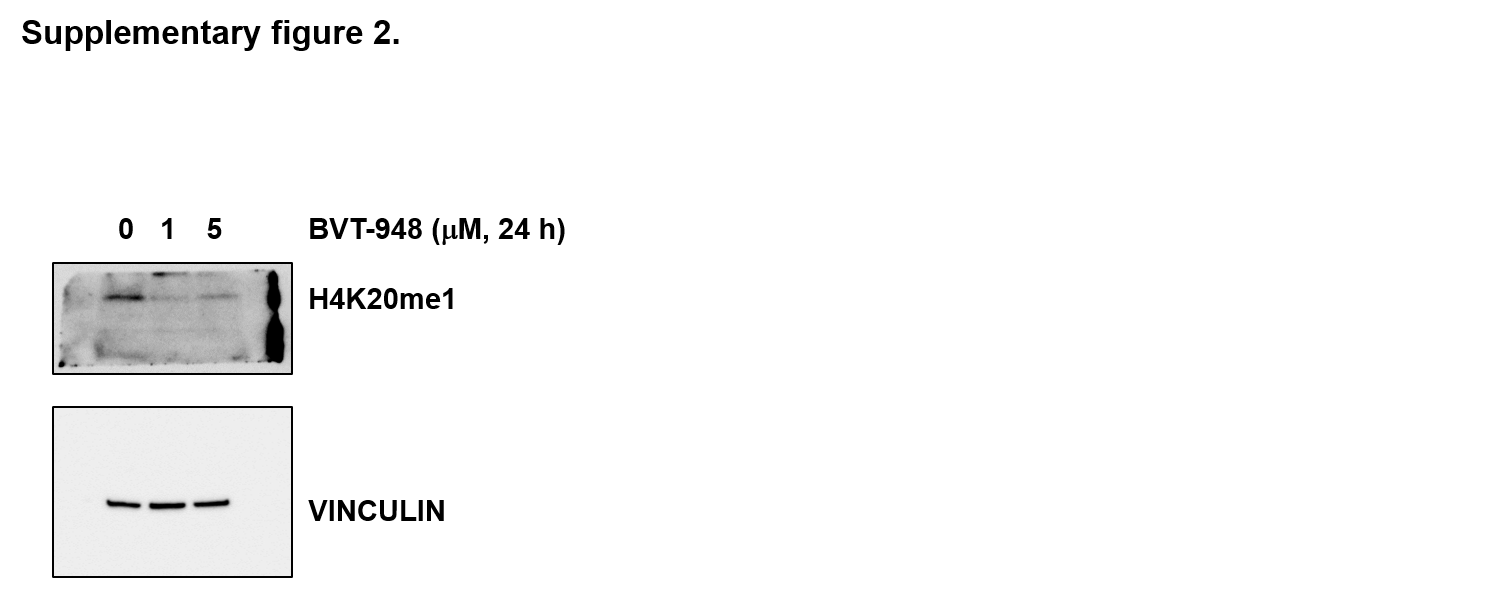


**Supplementary figure 4.** Full images of blots used in Figure 1A.


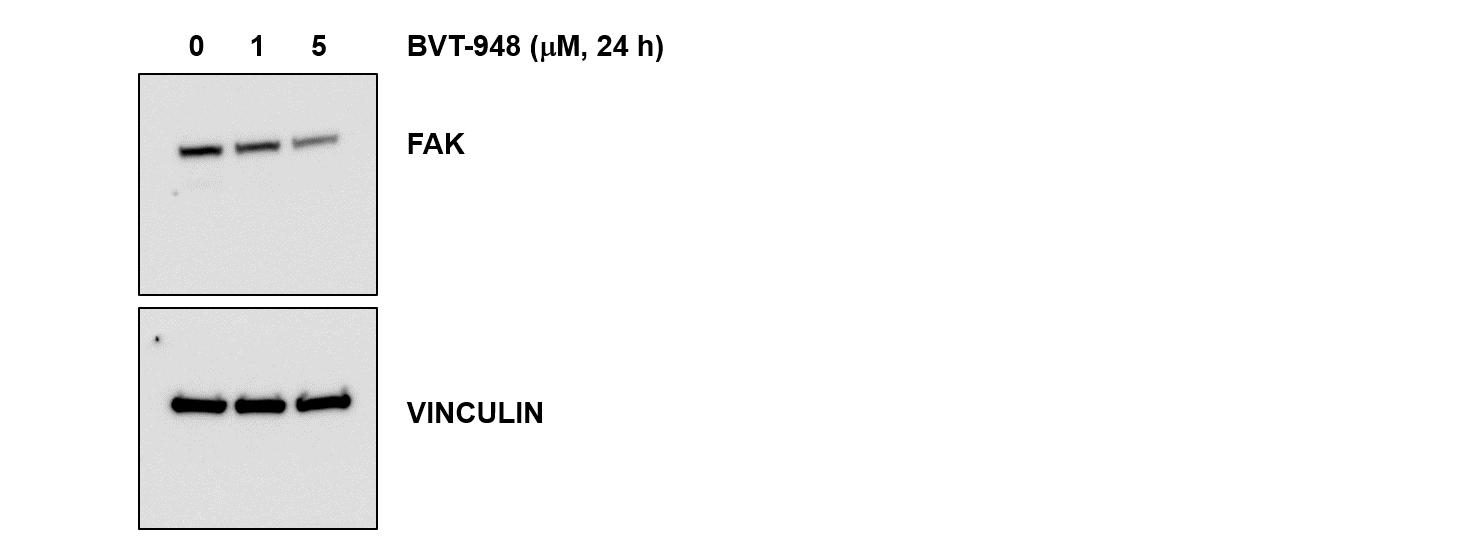


**Supplementary figure 5.** Full images of blots used in Figure 2 C.

**
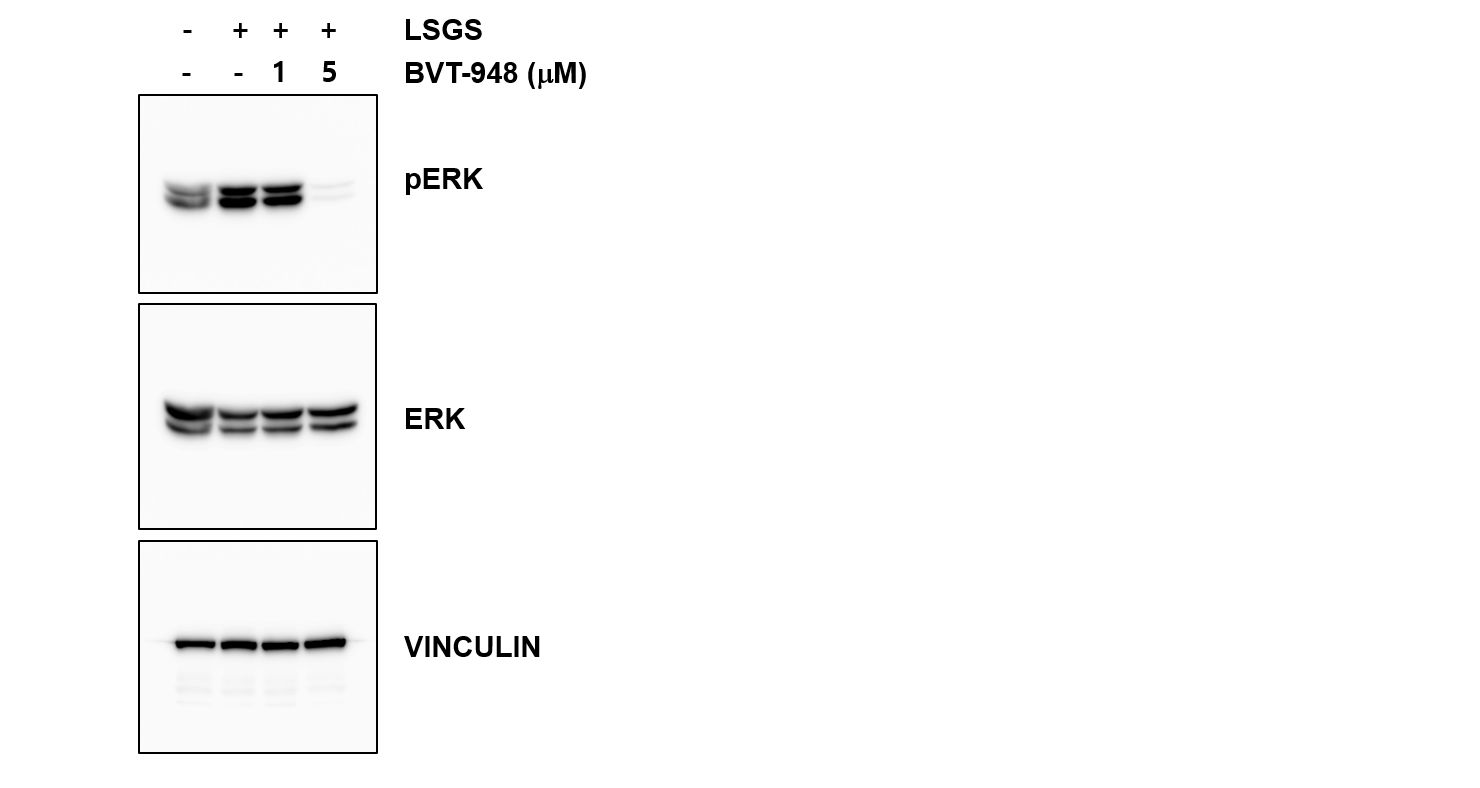
**

**Supplementary figure 6.** Full images of blots used in Figure 2 D.


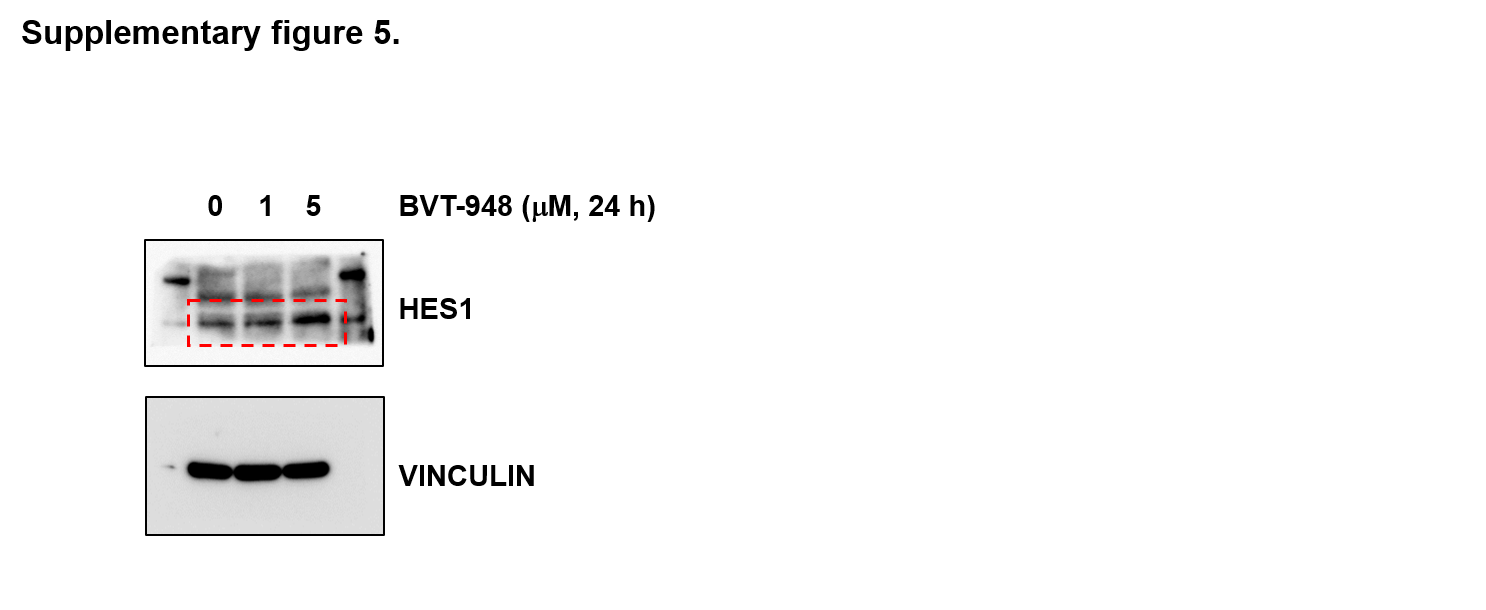


**Supplementary figure 7.** Full images of blots used in Figure 4 B. Dotted rectangles indicate the regions used in the figure.


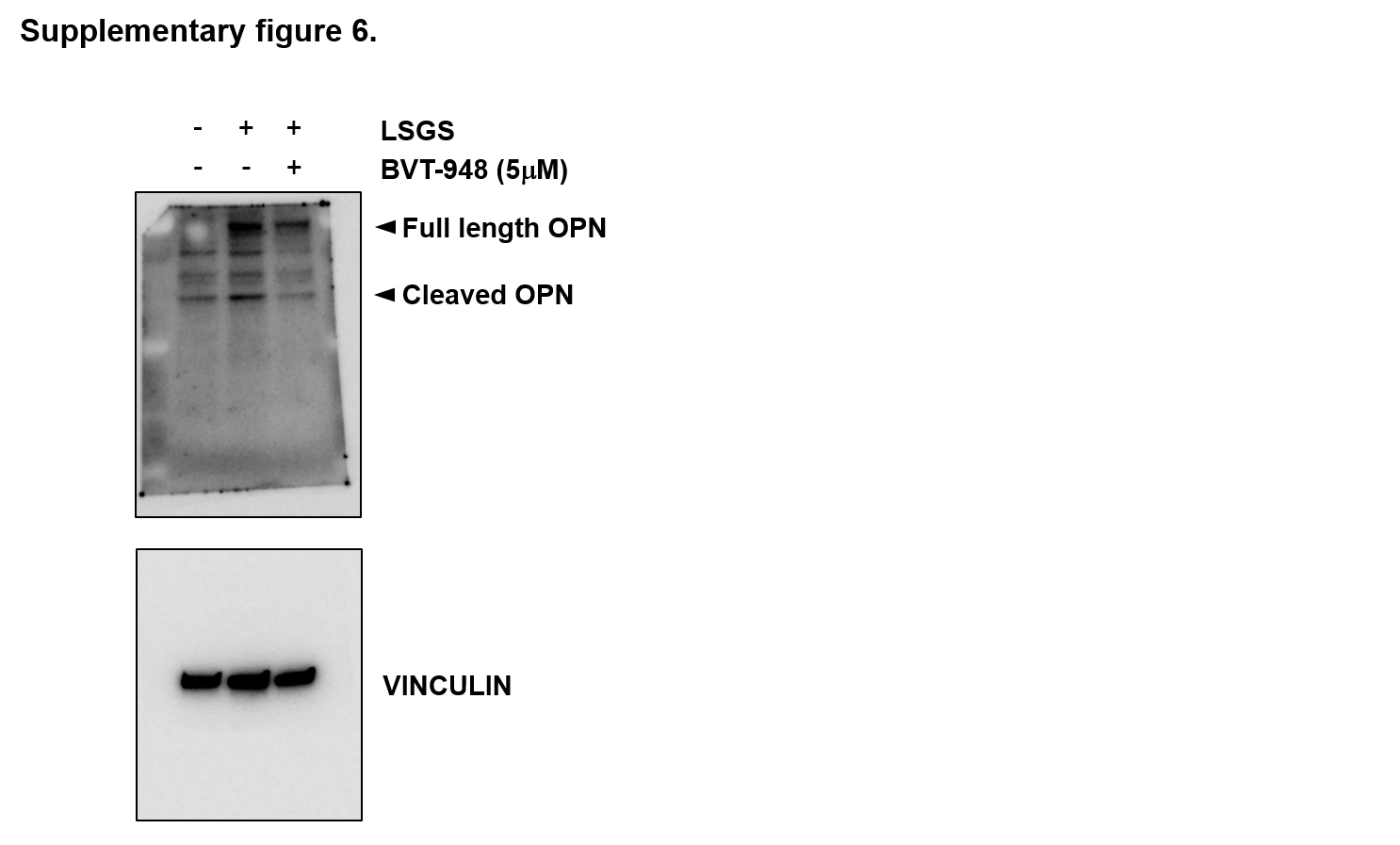


**Supplementary figure 8.** Full images of blots used in Figure 4C.


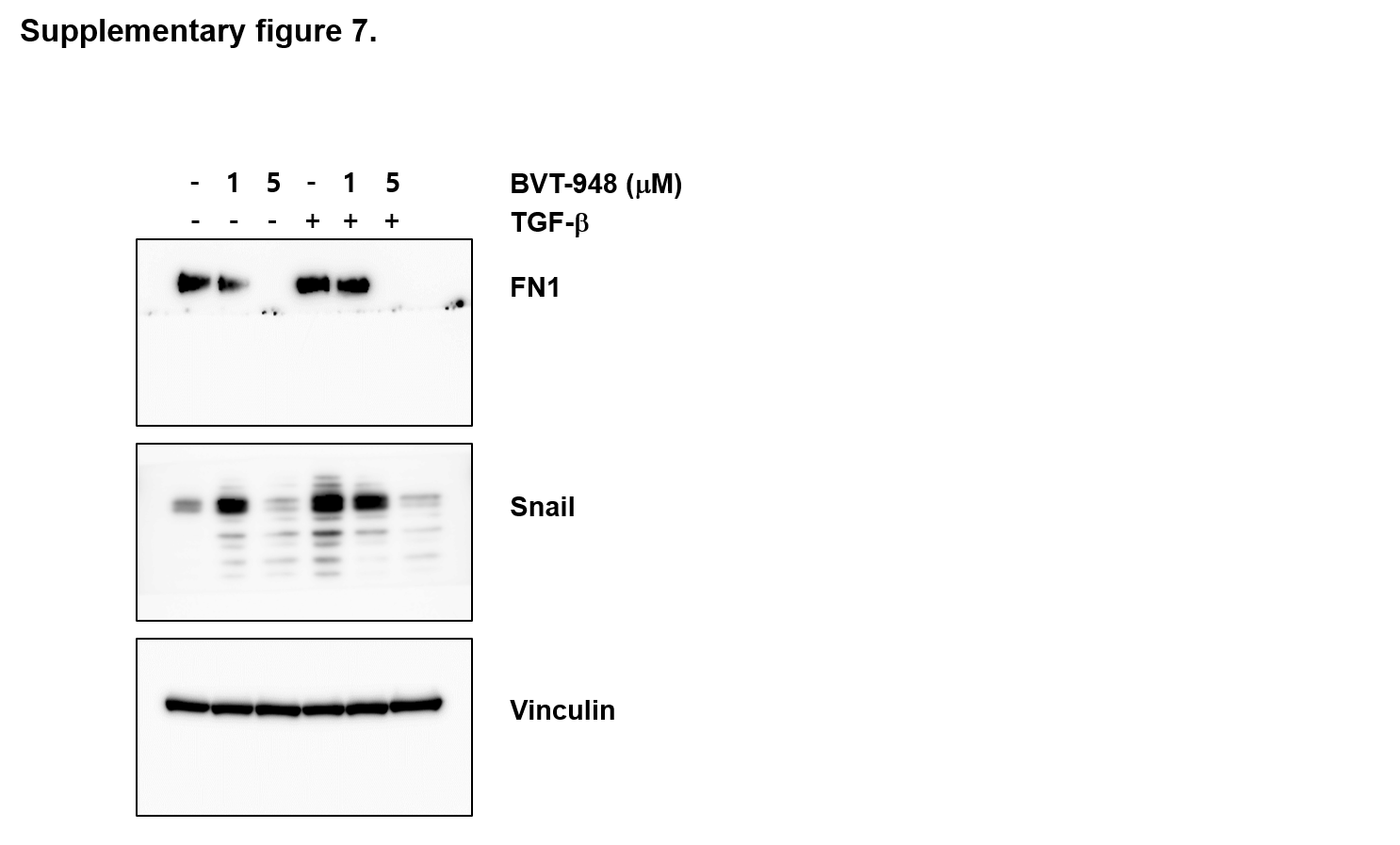


**Supplementary figure 9.** Full images of blots used in Figure 4E.

**Supplementary material & methods**

**Genetic ablation of SETD8 in hiPSCs by CRISPR/Cas9.**

To induce genetic mutation of SETD8 in hiPSCs, the targeting sgRNA template for the *SETD8* gene was synthesized by annealing and extension of two oligonucleotides (SETD8_sgRNA_primer_F: GAAATTAATACGACTCACTATAGGGCCTGCTTACCCCGTCGGTGGTTTTAGAGCTAGAAATAGCAAG, SETD8_sgRNA_primer_R: AAAAAAGCACCGACTCGGTGCCACTTTTTCAAGTTGATAACGGACTAGCCTTATTTTAACTTGCTATTTCTAGCTCTAAAAC). Then, *in vitro* transcription was performed by T7 RNA polymerase supplemented with NTPs and RNase inhibitor overnight at 37°C. *In vitro* transcribed sgRNAs were then treated with DNase I for 30 min at 37°C, and purified by using MinElute Cleanup kit (Quagen, Hilden, Germany). The CRISPR/Cas9 RNP complex was prepared by incubating 15 µg of Recombinant Cas9 (New England Biolabs, Ipswich, MA) and 10 µg of sgRNA at room temperatyre for 5min. To introduce Cas9 and sgRNA complex into hiPSCs, 2X10^5^ cells of hiPSCs were electroporated with Neon transfection system (Invitrogen) as manufacturer’s recommended parameter (pulse voltage: 1050v, pulse width: 30ms, and pulse number: 2 times). The transfected hiPSCs were cultured in mTeSR1 medium supplemented with 10µM of Y-27632 (STEMCELL technology).

**Differentiation of transfected hiPSCs into vascular endothelial cells.**

Wild-type or SETD8 RNP transfected hiPSCs were differentiated into vascular endothelial cells (Supplementary figure 1A). Briefly, 1X10^5 cells of hiPSCs was seeded on Matrigel-coated culture dish with mTeSR1 medium (STEMCELL technology). After 1 d, mTESR1 medium was changed to RPMI medium supplemented with 1% B27 supplement (Invitrogen), 50 ng/ml Activin A (Peprotech, Rocky Hill, NJ), 20 ng/ml BMP4 (Peprotech), and 1μM of CP21R7(Cayman chemical). After 3 d of culture, the cells were incubated in RPMI medium containing 50 ng/ml VEGF-A (Perprotech), and 50 ng/ml bFGF, and 2μM of Forskolin (Cayman chemical )for 2 days. After endothelial cell induction, the cells were cultured in EGM-2 medium (Lonza, Basel, Switzerland) supplemented with 10 ng/ml VEGF-A and 10 ng/ml bFGF for 3 days. Proportions of cells expressing respective endothelial markers(CD31 and CD144) were measured and analyzed using the BD FACS aria II the FlowJo software (Tree star, Ashland, OR), respectively.
